# Supplementary material for: Action sharpens sensory representations of expected outcomes
Source: Nat Commun. 2018 Oct 16;9:4288. doi: 10.1038/s41467-018-06752-7 (PMC6191413; doi:10.1038/s41467-018-06752-7)
Supplement: Supplementary file 1 — Supplementary Information [file 41467_2018_6752_MOESM1_ESM.pdf]

Action sharpens sensory representations of expected outcomes: Supplementary Information

Daniel Yon, Sam J. Gilbert, Floris P. de Lange & Clare Press

Supplementary Note 1: Prevalence analyses

We defined our ROIs using one-sample t-tests on decoding measures, which do not support population inference. This ROI-defining analysis might be seen as conceptually analogous to a functional localiser given that it aimed to identify where in our participants may be involved in processing observed stimuli, and is standard in the field. However, we wished to find out whether our main conclusions could be corroborated with a permutation test approach suggested by Allefeld *et al.*[1]. We investigated information prevalence by permuting class labels used for multivariate decoding (128 unique permutations), using the resultant decoding maps to create second-level permutations (1,000,000 random permutations) that were compared to our real, unpermuted data (see[1] for further details). We used the information prevalence approach to define voxels from which information about the observed stimuli was significantly prevalent (i.e. above chance in a majority of participants). In these voxels, decoding accuracy was higher for the congruent than incongruent condition ($F_{1,19}$ = 5.962, p= .025, η_p^2=.239), replicating the main analysis supporting the sharpening over the cancellation model (see Supplementary Fig.1A). Finally, these voxels also showed a congruency x stimulus preference interaction in the univariate signal ($F_{1,19}$ = 7.630 , p=.012, η_p^2=.287; see Supplementary Fig. 1B). Therefore, even using the approach suggested by Allefeld *et al.*[1] we still find the patterns of results that support the sharpening over the cancellation model.

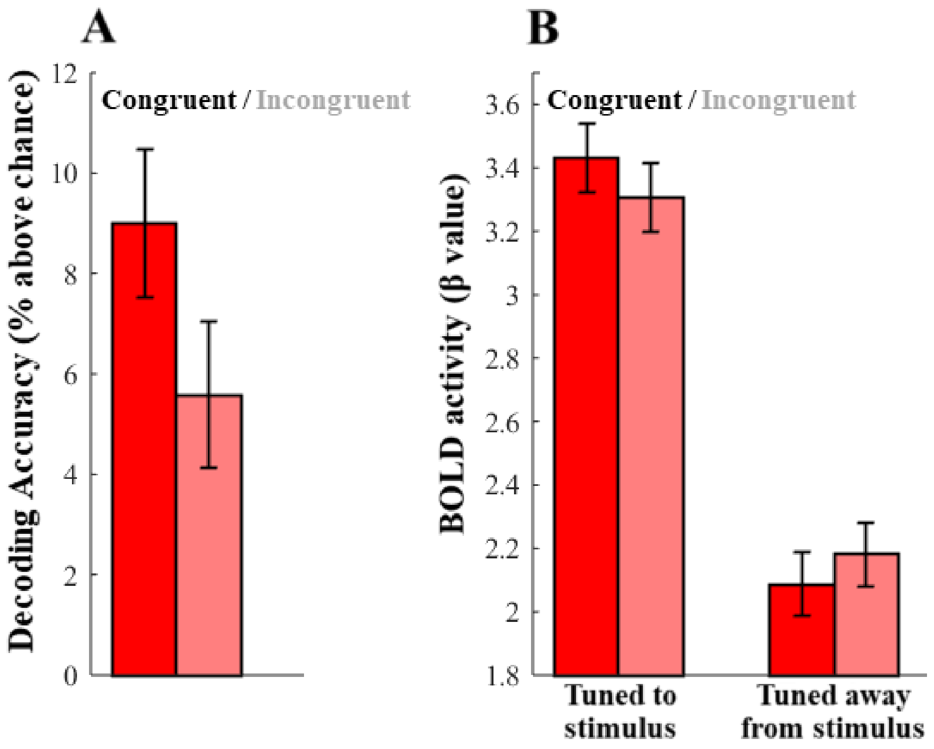

Supplementary Fig. 1. Stimulus decoding accuracies and univariate activity in voxels rejecting the majority null. A: Decoding accuracies on congruent (saturated) relative to incongruent (desaturated) trials. B: BOLD activity on congruent (saturated) relative to incongruent (desaturated) trials in voxels tuned to or tuned away from the current stimulus. Error bars show 95% within-participant confidence intervals of the mean difference between conditions $(N = 20, 95\%\ CI/\sqrt{2})$[5].

Supplementary Note 2: Univariate main effects of congruency

Sharpening models predict that suppression should be found in units tuned away from the expected stimulus. The fact that many (but not all, e.g.[2]) studies examining expectation mechanisms find a lower overall BOLD signal for expected stimuli therefore suggests that at the location of these previous effects there are a large number of voxels tuned away from, rather than towards, the expected stimuli. There was no evidence of an overall suppression of univariate activity for congruent action outcomes across our visual ROIs defined according to

where contained information about presented stimuli ($F_{1,19}$ = .625, p=.439, η_p^2=.032), likely because defining ROIs in this fashion will result in a greater number of voxels tuned to specific stimuli presented in this experiment, and by extension expected stimuli, relative to other approaches. Previous studies have often defined ROIs using functional localisers, but we did not incorporate subject-specific functional localisers given that we did not deem this appropriate for our primary aim. However, we examined whether there was an overall suppression of the signal on congruent trials at locations where these effects have been reported previously. We extracted univariate activity from voxels within a 6mm radius of the peak coordinates in left and right superior temporal sulcus in the studies with comparable designs[3,4] ([48,−42,18], [57,-57,17], [-56,-53,13]). We found weak evidence for lower overall activity on congruent relative to incongruent trials at these locations despite differences in participants and the specific stimuli used (t_{19} = 1.752, p< .05, one-tailed).

Supplementary references

1. Allefeld, C., Görgen, K. & Haynes, J.-D. Valid population inference for information-based imaging: From the second-level t-test to prevalence inference. *NeuroImage* **141,** 378–392 (2016).
2. Turk-Browne, N. B., Scholl, B. J., Chun, M. M. & Johnson, M. K. Neural evidence of statistical learning: efficient detection of visual regularities without awareness. *J. Cogn. Neurosci.* **21,** 1934–1945 (2009).
3. Kontaris, I., Wiggett, A. J. & Downing, P. E. Dissociation of extrastriate body and biological-motion selective areas by manipulation of visual-motor congruency. *Neuropsychologia* **47,** 3118–3124 (2009).
4. Leube, D. T. *et al.* The neural correlates of perceiving one's own movements. *NeuroImage* **20,** 2084–2090 (2003).
5. Loftus, G. R. & Masson, M. E. Using confidence intervals in within-subject designs. *Psychon. Bull. Rev.* 1, 476–490 (1994).
